# Specific Sizes of Hyaluronan Oligosaccharides Stimulate Fibroblast Migration and Excisional Wound Repair

**DOI:** 10.1371/journal.pone.0088479

**Published:** 2014-02-13

**Authors:** Cornelia Tolg, Patrick Telmer, Eva Turley

**Affiliations:** 1 London Regional Cancer Program, London Health Sciences Center, Victoria Hospital, London, Ontario, Canada; 2 Departments of Oncology, Biochemistry and Surgery, Schulich School of Medicine and Dentistry, Western University London, London, Ontario, Canada; National Institutes of Health, United States of America

## Abstract

The extracellular matrix polysaccharide hyaluronan (HA) plays a key role in both fibrotic and regenerative tissue repair. Accumulation of high molecular weight HA is typical of regenerative repair, which is associated with minimal inflammation and fibrosis, while fragmentation of HA is typical of postnatal wounds, which heal in the presence of inflammation and transient fibrosis. It is generally considered that HA oligosaccharides and fragments of a wide size range support these processes of adult, fibrotic wound repair yet the consequences of sized HA fragments/oligosaccharides to each repair stage is not well characterized. Here, we compared the effects of native HA, HA oligosaccharide mixtures and individual sizes (4–10mer oligosaccharides, 5 and, 40 kDa) of HA oligosaccharides and fragments, on fibroblast migration in scratch wound assays and on excisional skin wound repair *in vivo*. We confirm that 4–10mer mixtures significantly stimulated scratch wound repair and further report that only the 6 and 8mer oligosaccharides in this mixture are responsible for this effect. The HA 6mer promoted wound closure, accumulation of wound M1 and M2 macrophages and the M2 cytokine TGFβ1, but did not increase myofibroblast differentiation. The effect of 6mer HA on wound closure required both RHAMM and CD44 expression. In contrast, The 40 kDa HA fragment inhibited wound closure, increased the number of wound macrophages but had no effect on TGFβ1 accumulation or subsequent fibrosis. These results show that specific sizes of HA polymer have unique effects on postnatal wound repair. The ability of 6mer HA to promote wound closure and inflammation resolution without increased myofibroblast differentiation suggests that this HA oligosaccharide could be useful for treatment of delayed or inefficient wound repair where minimal fibrosis is advantageous.

## Introduction

Rapid and efficient wound repair is crucial for infection prevention but can come at the expense of scar formation due to tissue fibrosis. Fibrotic healing that results in scar formation has a significant negative socioeconomic impact due to loss of tissue function and psychosocial morbidity [1], [2], [3]. Therefore, strategies restoring or optimizing wound repair efficiency without increasing wound fibrosis are desirable.

Fibrotic repair is a process characterized by inflammation, wound contraction, excessive accumulation and cross-linking of collagen and neo-angiogenesis. In contrast, mid-gestation fetal wounds heal by a “scarless” or regenerative type of repair in the absence of fibrosis [1], [4], and this is characterized by an attenuated inflammatory response, increased Collagen III to Collagen I ratios, reduced TGFβ 1 but increased TGFβ 3 levels and, of relevance to the present study, increased and prolonged accumulation of high molecular weight (MWavg>1 million Da) hyaluronan (HA) but reduced HA fragmentation.

HA is a glycosaminoglycan consisting of repeating disaccharide units of N-acetylglucosamine and glucuronic acid, which is increased during repair of both fetal and adult skin. In fetal skin, HA remains largely as a native high molecular weight polymer while in adult skin it is degraded into millions of different sizes as a result of both the enzymatic activity of hyaluronidases (HAdase) and reactive oxygen/nitrogen species (ROS/NOS) [5], [6], [7], [8]. HA fragmentation has been shown to stimulate key aspects of fibrotic wound repair including wound contraction, inflammation, neo-angiogenesis, fibroplasia, myofibroblast differentiation and increased collagen production/crosslinking. Indeed, if HA fragments and oligosaccharides are not removed from injured tissues, unremitting inflammation and tissue destruction resulting from uncontrolled fibrosis ensures [9]. In contrast, high molecular weight native HA dampens the fibrotic process by attenuating inflammation and fibroplasia [1], [10], [11], [12].

HA mediates its effects through cell surface receptors such as RHAMM/HMMR, CD44, LYVE1 and TLR2,4, which activate downstream signaling pathways including PI3 kinase/AKT and MEK1/ERK1,2. Absence of these HA receptors has profound effects on skin wound repair. For example, repair of excisional skin wounds is aberrant and delayed in RHAMM−/− mice due to blunted inflammation, angiogenesis, mesenchymal cell migration and fibroplasia [13]. Expression of a CD44 antisense construct in keratinocytes reduces wound re-epithelialization, keratinocyte proliferation and inflammation [14]. TLR4 is expressed by keratinocytes at wound edges during early stages of wound repair and absence of TLR4 results in delayed repair of excisional wounds and blunted IL-6 and IL-1beta production in wounds [15].

Studies over the past several decades have established that native HA has different effects on cells than its fragmented counterparts, however, the mechanisms responsible for this size dependency are poorly understood and it is not yet clear if fragment bioactivity resides in a range of sizes or is size-specific. For example, several reports in this understudied field identify ranges of polymer sizes that exhibit differential effects on wound repair. Intermediate sized HA fragments (100–300 kDa) promoted scratch wound closure by human keratinocytes while smaller fragments (5–20 kDa) did not [16], and 50–400 kDa but not <50 kDa HA fragments promoted keratinocyte proliferation and epidermal hyperplasia [17]. Moreover, a heterogeneous mixture of small HA oligosaccharides (4–20mers, 0.8–4 kDa) increased angiogenesis and repair of excisional wounds [18]. These studies did not address the possibility that only specific HA polymer sizes within the range tested are responsible for the effects on wound repair. Defining the size dependent effects of HA fragments more precisely will greatly increase understanding of the molecular mechanisms responsible for tissue fibrosis.

Isolating single molecular weight polymers in the above size ranges has historically been technically challenging. However, HA oligosaccharides are more easily separated into distinct polymer sizes than larger HA fragments, and large amounts of single species of HA oligosaccharide can be biochemically generated by recombinant synthases. Since we, and others, have shown that a mixture of 4–24mer HA promotes migration in scratch wound assays [8], [19], we chose this oligosaccharide size range to test our hypothesis that bioactivity of HA is precisely size dependent. For our analyses we compared the effects of HA oligosaccharide mixtures with individual oligosaccharides, native HA (500 kDa) and sized HA fragments (5 and 40 kDa) on dermal fibroblast migration in scratch wounds in culture. We then tested the effect of the HA sizes that affected cell migration *in vitro* on wound closure, inflammation and fibrosis (myofibroblast differentiation) *in vivo*.

We confirmed that HA oligosaccharide mixtures promoted fibroblast migration in scratch wound assays, the larger HA fragments lacked this activity and native HA inhibited migration. We showed that only the 6 and 8mers present in the HA oligosaccharide mixture had migration-enhancing activity in culture. *In vivo* analyses showed that the 6mer stimulated wound closure, increased M1 and M2 macrophages and resulted in an elevation of wound TGFβ1 accumulation. These significant changes did not result in increased wound fibrosis as detected by changes in smooth muscle actin or collagen staining. Our results thus suggest a model whereby individual HA oligosaccharides have distinct biological properties and further predict that different sizes of HA are required to complete the fibrotic process: A 6mer is sufficient to increase macrophage infiltration and TGFβ1 expression but not to promote myofibroblast differentiation. Our results further predict that topical application of 6mer HA fragments may be of therapeutic use to stimulate or accelerate wound repair without increasing wound fibrosis.

## Materials and Methods

### Materials

Purified HA oligosaccharides were obtained from Hyalose (Oklahoma City, OK, USA). The 5 kDa, 40 kDa and 500 kDa HA preparations were purchased from Lifecore Biomedical, LLC (Chaska, MN, USA) and have a molecular weight range of <10 kDa, 41–65 kDa and 351–600 kDa, respectively. All HA preparations were free of DNA and protein contaminations. A mixture of HA fragments and oligosaccharides was produced by incomplete digestion of polydisperse 240 kDa HA (Hyal Pharmaceutical Corp.; Mississauga, Canada) with Streptococcus hyaluronidase (Sigma-Aldrich Canada, Oakville, ON, Canada) to generate a heterogeneous mixture of HA fragments (MWav 10 kDa) as previously described [8].

Antibodies were used at a dilution recommended by the manufacturer. Cell culture inserts for scratch wound assays were purchased from ibidi Gmbh (Martinsried, Germany). Masson’s Trichrome staining kit was obtained from Sigma-Aldrich. Sprague-Dawley rats were purchased from Charles –River (Sherbrooke, Canada) and rat dermal fibroblasts were obtained from ATCC. RHAMM−/− and CD44−/− mice were bred in house and are described elsewhere [20], [21]. Smooth muscle actin antibodies were purchased from Sigma-Aldrich (Oakville, ON, Canada), iNOS, ARG, Tenascin C, TBFβ1 (active and non active form) antibodies were purchased from Abcam Inc. (Toronto, ON,Canada). Secondary antibodies and non-immune IgG were purchased from Jackson Laboratories Inc. (West Grove, PA, USA).

### Postnatal Excisional Wound Repair

All animal experiments were approved (protocol # 2009-060) by the animal use committee of Western University following Canadian Council of Animal Care guidelines. 6 weeks old female Sprague-Dawley rats were anesthetized using isoflurane inhalation. Once animals were non-conscious, hair on back was removed using an electric razor. Three full thickness punch biopsies were taken along the midline of the back using a 4 mm metal punch creating 6 full thickness excisional wound. Wounds were filled with 100 µl collagen I matrix mixed with either HA (1–50 µg/ml) or PBS. Animals were kept under anesthesia for another 30 min to allow collagen to form a gel. Animals were housed individually for the duration of the experiment to avoid interference with wound repair by grooming and fighting.

### Wound Repair Quantification

Animals were anesthetized every second day and wound edges were traced on a plastic sheet. Tracings were digitized by scanning and wound areas were quantified using Image J software (National Institutes of Health http://imagej.nih.gov/ij).

### Histology

Animals were anesthetized at the indicated time points and wounds were harvested using an 8 mm metal punch. Following this procedure animals were sacrificed by exsanguinations under anesthesia. Wound tissue was fixed in 3.7% Paraformaldehyde pH 7.4 and embedded in paraffin. 10 µm sections were cut from the centre of the wound. Sections were de-paraffinized in Xylenes (2×10 min) and re-hydrated by passage through a series of decreasing ethanol concentration (100%, 96%, 70%, 0% 5 min each). Antigens were retrieved by 20 min boiling in 0.1 mM pH 6 NA-Citrate using a microwave. Endogenous peroxidase was blocked by incubating sections for 10 min in 3% H_2_O_2_/PBS. Non specific binding was blocked by incubating sections in 2% BSA/PBS for 1 hr at RT. Sections were incubated with primary antibodies that were diluted in 1% BSA/PBS over night at 4°C. Control sections were incubated with non immune IgG. Sections were washed 3×5 min in PBS and then incubated with secondary antibodies that were diluted in 1% BSA/PBS for 1 hr at RT. Sections were washed 3x with PBS and peroxidase was detected with DAB following manufacturer’s instructions. Sections were counterstained with hematoxylin and mounted using Cytoseal 40. Sections were digitized by scanning and staining was quantified by either analyzing area (pixels) that was stained above an arbitrary background using ImageJ software or by counting positively stained cells/tissue area. Colour deconvolution plugin of Image J was used to separate blue Hematoxylin and brown DAB staining before setting a threshold for DAB staining intensity to be considered above background.

### Modified Scratch wound Assays

Rat dermal fibroblasts were obtained from ATCC and cultured in DMEM, 10% FBS, Antibiotic-antimycotic at 37°C, 5% CO_2_, water saturated atmosphere. Cells were seeded at high density in ibidi culture inserts. The following day, culture inserts were removed and medium was changed to DMEM, 1% FBS plus HA. Images were taken every 6 hrs to follow migration of cells into the cell free space. Cell migration was quantified by counting cells that had migrated into the cell free space at a certain time/unit area.

Statistical analysis: results were analyzed by ANOVA and Student’s ttest. P values <0.05 were considered to be significant.

## Results

### Specific Sizes of HA Stimulate Rat Dermal Fibroblast Migration

**Figure 1:**
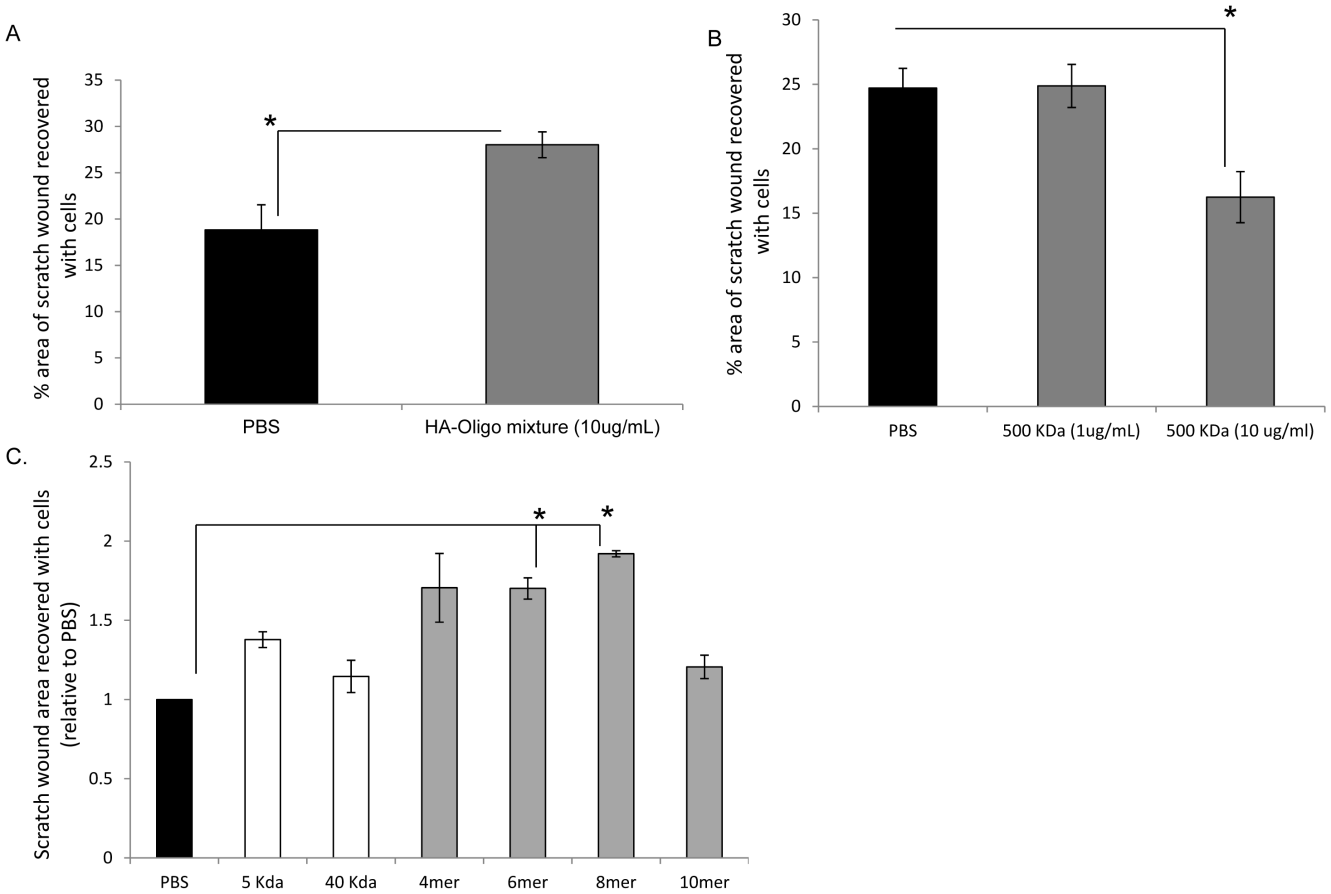
6 and 8mer HA fragments stimulate but HMW HA inhibits rat dermal fibroblast migration in modified scratch wound assays. Migration of rat dermal fibroblasts was analyzed using Ibidi culture inserts as described in Material and Methods. A: Mixture of HA oligomeres significantly stimulate migration of rat dermal fibroblasts. Cells were treated with DMEM 1% FBS containing a HA oligomere mixture consisting of equal parts of 4mer, 6mer, 8mer and 10mer HA at a final concentration of 10 µg/ml. Cell migration was quantified by measuring the area of cells that migrated into the empty space after removal of the culture insert. Graph represents Mean±SE of N = 6 culture wells B: HMW HA inhibits migration of rat dermal fibroblasts. Cells were treated with DMEM 1% FBS containing 10 µg/ml 500 kDa HA. Cell migration was quantified by measuring the area of cells that migrated into the empty space after removal of the culture insert. Graph represents Mean±SE of N = 6 culture wells C: 6 and 8mer significantly stimulate migration of rat dermal fibroblasts. Cells were treated with DMEM 1% FBS containing either 4mer, 6mer, 8mer, 10mer, 5 kDa, 40 kDa HA at 1 µg/ml or PBS over night. Cell migration was quantified by measuring the area of cells that migrated into the empty space after removal of the culture insert. Graph represents Mean±SE of N = 4 culture wells.

Excisional skin wounds in rats contain HA oligosaccharides and fragments ranging from 4mer-500 kDa [8]. Topical application of mixtures of 6–20mer HA fragments have been reported to augment the repair of full thickness excisional wounds by stimulating angiogenesis, lymph-angiogenesis and fibroplasia [19] [18]. This same mixture of HA fragments stimulate scratch wound induced migration of endothelial cells in culture suggesting that increased cell migration is at least partly responsible for the augmented angiogenesis observed *in vivo* [19]. Furthermore, heterogeneous mixtures of low molecular weight HA fragments and oligosaccharides (10 kDa avg. MW) have previously been shown to stimulate scratch wound-induced migration of dermal fibroblasts [8]. We therefore initially compared the effects of a similar mixture of HA oligosaccharide sizes, with individual sizes of HA oligosaccharides contained in the mixture, as well as HA fragments (5 kDa and 40 kDa) and native HA (500 kDa) on rat dermal fibroblast migration into scratch wounds. As predicted, a mixture of equal amounts of 4, 6, 8, and 10mer HA fragments (p<0.05, Fig. 1A) or a 10 kDa MWav HA generated by incomplete digestion with *Streptococcus* hyaluronidase (data not shown) stimulated cell migration at a concentration of 10µg/ml. In contrast, the 5 kDa and 40 kDa HA fragments had no significant effect on migration, and high molecular weight HA (500 kDa) significantly (p<0.05) inhibited migration of rat dermal fibroblasts when used at the same concentration as the HA oligosaccharide mixtures (10 µg/ml) (Fig. 1B,C). To investigate which of the HA oligosaccharide sizes in the 4–10mer mixture stimulated migration, we separately added the 4, 6, 8, and 10mer HA to scratch wounded cultures of rat dermal fibroblasts. Only the 6mer and 8mer HA fragments significantly stimulated (1.7–1.9 fold, p<0.05) migration of rat dermal fibroblasts at 1 µg/ml relative to the PBS control (Fig. 1C). Notably, the 4 and 10mer oligosaccharides had no significant effect on migration of these cells (Fig. 1C) although the 4mer HA clearly had variable activity which did not reach statistical significance. This may be due to a suboptimal ability of this size to interact with and activate HA receptors required for cell migration. Collectively, the results suggested that migration-stimulating activity for dermal fibroblasts is restricted to a specific size (6 and 8mer) of HA oligosaccharide, and this property is not shared by native (500 kDa) HA, or HA fragments (5 and 40 kDa).

### 6mer HA Oligosaccharide Stimulates Closure of Full Thickness Excisional Wounds

**Figure 2:**
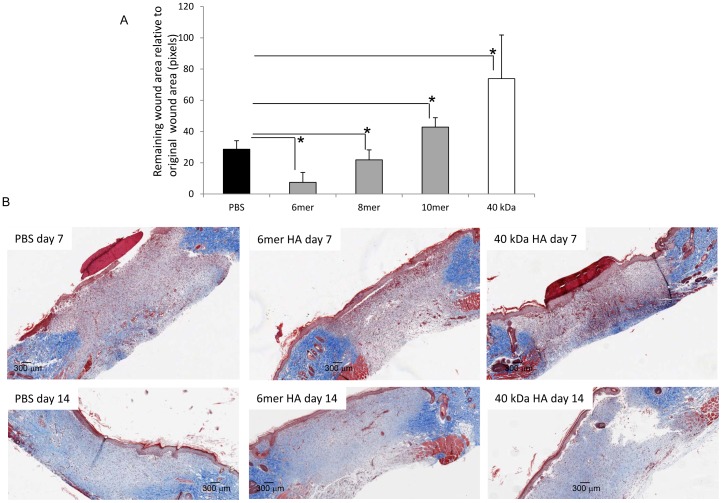
6mer HA fragments accelerate repair of full thickness excisional wounds. Full thickness excisional wounds were treated with a mixture of Collagen I and either 6mer, 8mer or 10mer 40(1–50 µg/ml) or PBS as described in Material and Methods. A: Wound edges were traced and remaining wound area quantified by image analysis. Graph shows remaining wound area relative to original wound size on day 7 after wounding. Mean±SE of N = 6 wounds. B: Wound closure is completed 2 weeks after wounding. Representative images of 2 weeks old wounds that were stained with Masson’s Trichrome are shown.

During repair of excisional wounds, dermal fibroblasts must migrate from wound edges into the provisional matrix of the granulation tissue in order to participate in wound closure. This provisional matrix contains a highly polydisperse population of native HA as well as HA fragments and oligosaccharides. A subpopulation of these fibroblasts differentiates into myofibroblasts in order to significantly complete postnatal wound closure and repair/wound resolution. Since the 6 and 8mer HA fragments uniquely stimulated migration of rat dermal fibroblasts in culture, we reasoned that these oligosaccharides might also be critical for the fibroblast migration into wounds that is part of the process of closing excisional wounds *in vivo*. We therefore measured the effect of different HA sizes on closure of excisional skin wounds. We assessed this by comparing the effect of these two HA sizes with those (e.g. 10mer and 40 kDa) that did not affect migration on wound closure. For these experiments, we treated full thickness excisional wounds with different HA preparations (6mer, 8mer 10mer HA fragments or 40 kDa HA at a concentration of 1–50 µg/ml) mixed with Collagen I to retain the fragments at the wound site, and compared closure of the wounds (Fig. 2). Wound repair was quantified by macroscopically tracing wound edges on day 0, 3, 5 and 7 followed by quantification of the remaining wound area by image analysis software (Image J) (Fig. 2A). Wounds treated with only the 6mer HA closed more rapidly (3.8 fold, p<0.05) than wounds treated with PBS/collagen I alone. The 8mer also significantly stimulated wound closure (1.3 fold p<0.05) but the effect was less than seen with the 6mer. In contrast, the 10mer HA and 40 kDa fragments significantly inhibited closure (10mer 1.5 fold p<0.05, 40 kDa 2.6 fold p<0.05). Masson’s Trichrome staining of wound cross sections revealed that all wounds were fully repaired and had resolved after 2 weeks (Fig. 2B). Excisional wound closure *in vivo* is obviously much more complex than gap closure in mono-culture but our results suggest that the 6mer HA has unique functional properties in enhancing wound closure including an ability to promote fibroblast migration. Our results also predict that build up of larger HA oligosaccharides and fragments in wounds can delay wound closure.

In addition to fibroblast in-migration, closure of postnatal excisional wounds is facilitated by inflammation, myofibroblast differentiation and extracellular matrix remodeling. We therefore next assessed if any of these properties were modified by application of the 6mer HA oligosaccharide.

### 6mer HA Significantly Increases Wound TGFβ1 Accumulation

**Figure 3:**
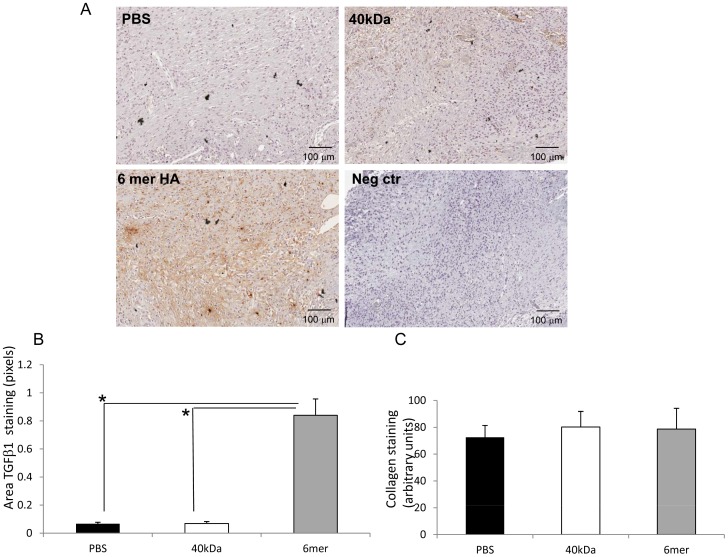
6mer HA significantly increases TGFβ1 accumulation but has no effect on collagen accumulation during wound repair. A, B: Cross sections of 7 days old wounds were stained with TGFβ1 specific antibodies as described in Materials and Methods. Positive stained area was quantified by image analysis using ImageJ. Graph shows Mean±SE of N = 18 (6 sections, three areas/section). C: Cross sections of 7 days old wounds were stained with Masson’s Trichrome as described in Materials and Methods. Blue staining (Collagen) was quantified by image analysis using ImageJ. Graph shows Mean±SE of N = 18 (6 sections, three areas/section).

A number of cytokines that promote wound closure are present in the blood that bathes the wound. One of these, TGFβ1 has also particularly been linked to HA-regulation functions including fibroblast and macrophage migration/polarization [22], and myofibroblast differentiation [4], [23], [24]. We next assessed if the 6mer HA oligosaccharide affected the amount of TGFβ1 found in treated vs. untreated wounds. We also analyzed the affect of the 40 kDa HA fragment on wound TGFβ1 accumulation since this had reduced wound closure, and might be expected to reduce TGFβ1 accumulation. 7 day old wounds that were either treated with one topical application of collagen I matrix mixed with either 6mer HA or 40 kDa HA were stained for TGFβ1 using IHC. Wound TGFβ1 was strongly and significantly (p<0.05) increased in response to 6mer HA compared to the PBS control (Fig. 3 A, B). In contrast, the 40 kDa fragment had no significant effect on TGFβ1 accumulation. Since TGFβ1 is produced by macrophages and is an agonist for M2 polarization, required for ECM remodeling, myofibroblast differentiation and wound resolution, we next assessed if the increase in wound TGFβ1 effected by the 6mer translated into detectable changes in wound macrophage populations.

### 6mer HA Significantly Increases Wound M1 and M2 Macrophage Infiltration

The infiltration of M1 or classically activated macrophages into wounds is an important initiating event in early pro-inflammatory wound repair stages of postnatal skin. These macrophages aid in infection prevention but also produce numerous cytokines and growth factors that set up the subsequent, fibrotic stages or repair including fibroplasia, myofibroblast differentiation and wound remodeling/resolution [25]. Thus, M1 macrophages are responsible for sustaining sufficient inflammation to provoke a profibrotic response. In contrast the paracrine factors derived from M2 or alternatively activated macrophages attenuate early inflammation and promote fibroplasia and collagen production associated with the fibrosis stage of wound repair and resolution [26]. Wound TGFβ1 is an agonist for M2 polarization of wound macrophages and is also characteristically produced by these macrophages. Consequently, relatively high numbers of wound M1 macrophages are indicative of a strong inflammatory reaction and high levels of M2 macrophages associated with elevated TGFβ1 accumulation generally predict a robust fibrotic repair.

**Figure 4:**
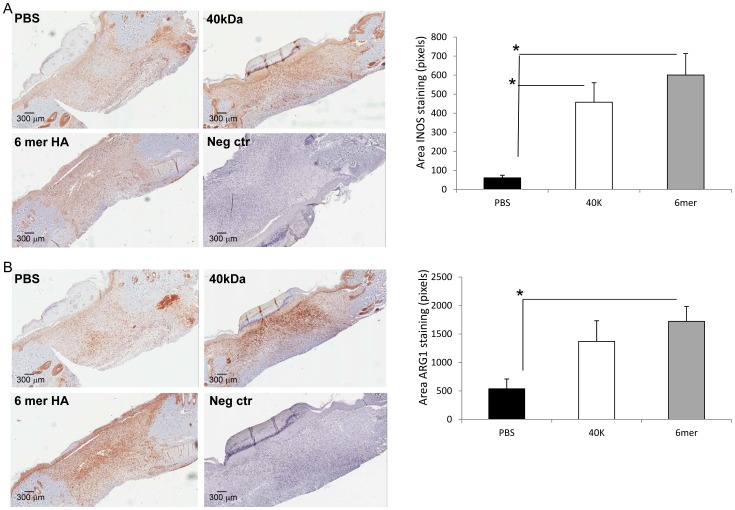
6mer and 40 kDa HA moderately increase macrophage infiltration during wound repair. A: Cross sections of 7 days old wounds were stained with INOS specific antibodies as described in Materials and Methods. Positive cells were counted/area granulation tissue. Graph shows Mean±SE of N = 18 (6 sections, three areas/section). B: Cross sections of 7 days old wounds were stained with ARG1 specific antibodies as described in Materials and Methods. Positive cells were counted/area granulation tissue. Graph shows Mean±SE of N = 18 (6 sections, three areas/section).

The number of wound M1 and M2 macrophages in 6mer and 40 kDa HA treated 7 day wounds were compared to PBS controls and identified using INOS (M1) and ARG1 (M2) specific antibodies (Fig. 4 A,B). The infiltration of both M1 and M2 macrophages was significantly increased in wounds treated with 6mer HA vs. PBS controls (Fig. 4 A,B). These results indicate that a robust inflammatory process was promoted by the 6mer HA oligosaccharide and that the pro-fibrotic M2 macrophages were also increased, consistent with the high accumulation of wound TGFβ1. Since this cytokine is an agonist for M2 polarization, these results further suggest that at least some of the 6mer HA-stimulated TGFβ1 is in its active form. Similar to 6mer HA, 40 kDa HA increased infiltration of M1 and M2 macrophages. However, whereas this effect on M1 macrophages was statistically significant the increase in M2 macrophage infiltration did not reach statistical significance (Fig. 4A,B p<0.05).

### HA Fragments do not Detectably Affect Later Stages of Fibrotic Repair in Wounds

**Figure 5:**
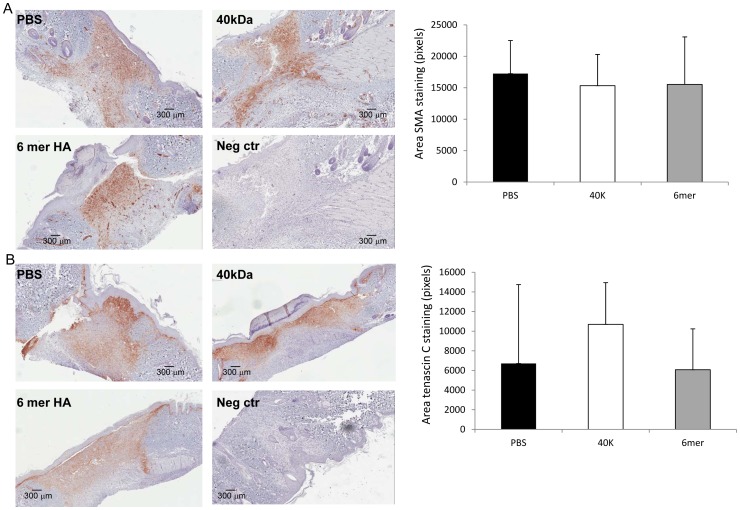
6mer HA and 40 kDa HA has no significant effect on Smooth muscle actin or Tenascin C expression during wound repair. A: Cross sections of 7 days old wounds were stained with Smooth muscle actin specific antibodies as described in Materials and Methods. Positive stained area was quantified by image analysis using ImageJ. Graph shows Mean±SE of N = 18 (6 sections, three areas/section). B: Cross sections of 7 days old wounds were stained with Tenascin C specific antibodies as described in Materials and Methods. Positive stained area was quantified by image analysis using ImageJ. Graph shows Mean±SE of N = 18 (6 sections, three areas/section).

The above results indicate that both the 6mer and 40 kDa HA fragment contain pro-inflammatory activity but that the 6mer uniquely promotes very early stages of tissue fibrosis, as indicated by the increased presence of TGFβ1 and M2 macrophages. A robust fibrotic repair is also characterized by angiogenesis, myofibroblast differentiation, increased collagen production and evidence of ECM remodeling that is best observed in rodent excisional skin wounds at 7 days post-injury [8], [13]. This time point was therefore examined for evidence of increased fibrosis in response to the 6mer. PBS and 40 kDa HA fragments were used as controls. Since TGFβ1 was strongly elevated in wounds following 6mer HA treatment and this cytokine is necessary for myofibroblast differentiation through an HA-mediated mechanism [27], we quantified myofibroblast differentiation using smooth muscle actin staining and elevated wound collagen as markers. Surprisingly, neither smooth muscle actin staining nor wound collagen levels were increased by treatment with the 6mer oligosaccharide (Figs. 3C, 5A). The 40 kDa fragment also had no effect relative to the PBS control (Figs. 3C, 5A). Wound angiogenesis was quantified by counting the number of smooth muscle actin-positive blood vessels but this parameter was also not altered in HA treated (6mer or 40 kDa) vs. PBS control wounds (data not shown). Evidence for ECM remodeling was also quantified using tenascin C staining as a marker. This ECM protein has also been used as a marker for wound regeneration [28]. As shown in Fig. 5B, neither the 6mer nor 40 kDa HA fragment promoted accumulation of tenascin C above that of the PBS control. Collectively, these results suggest that, although the 6mer HA oligosaccharide enhanced fibroblast migration, wound closure, TGFβ1 accumulation, inflammation (marked by M1 macrophages) and early stages of fibrosis (marked by M2 macrophages and elevated TGFβ1), this did not result in enhancements of later stages of fibrosis.

### RHAMM and CD44 are both Required for Stimulation of Wound Repair by 6mer HA

**Figure 6:**
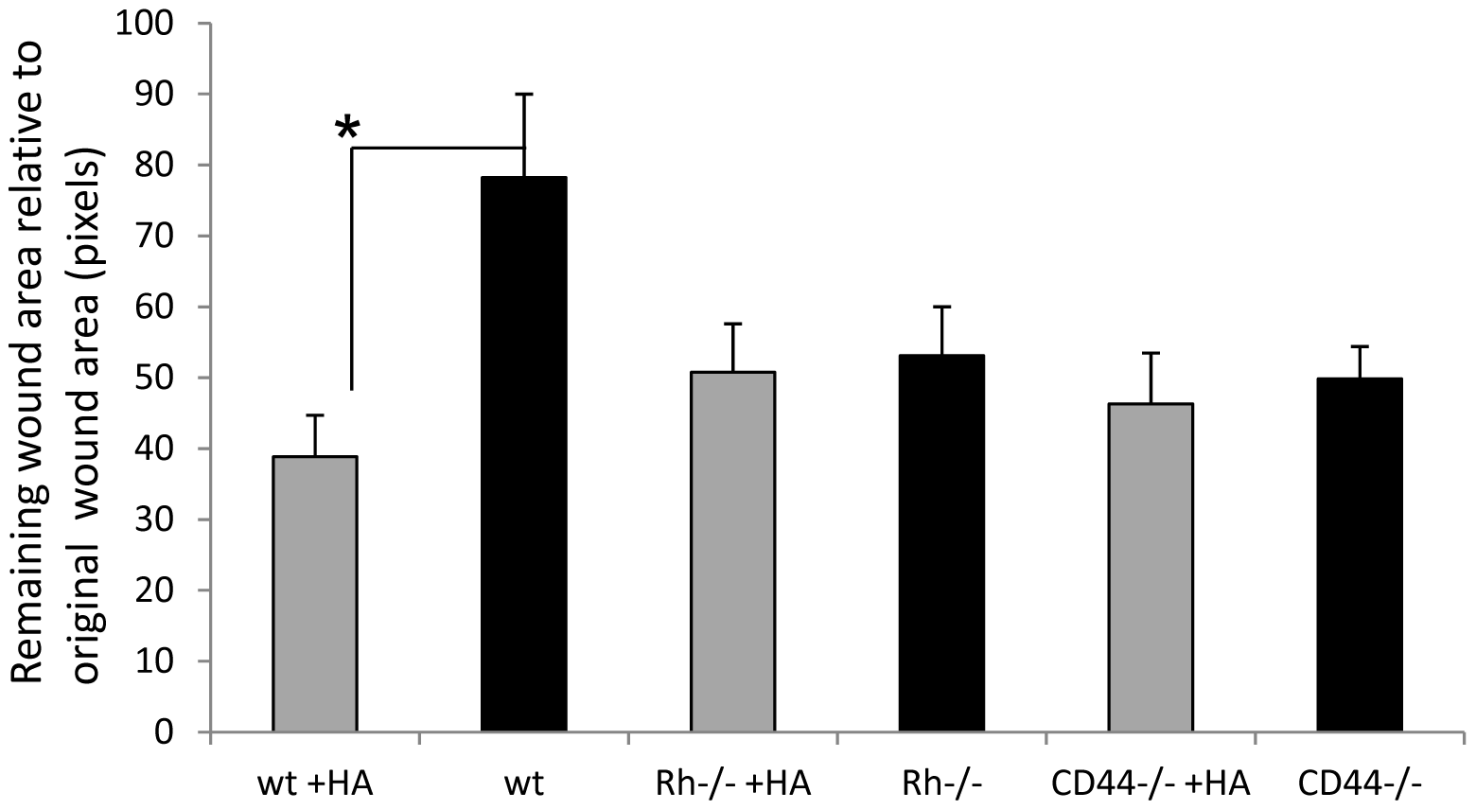
RHAMM and CD44 are both required for stimulation of wound repair by 6mer HA. Wild type (wt), CD44−/− and RHAMM−/− (Rh−/−) were wounded as described in Material and Methods. Wounds were treated with Collagen I +/−6mer HA as described in Material and Methods. Wound edges were traced and remaining wound areas quantified by image analysis. Graph shows remaining wound area relative to original wound size on day 5 after wounding. Mean±SE of N = 6 wounds.

Efficiency of wound repair is determined by complex interplay between diverse cell types such as keratinocytes, fibroblasts, endothelial cells, macrophages and lymphocytes, all of which express HA receptors and therefore have the potential to interact with and respond to 6mer HA. Typically, RHAMM and CD44, which have previously been associated with wound repair, are expressed on most skin cells present in wounds while TLR2,4 are expressed by pro-inflammatory innate immune cells and LYVE1 is mainly expressed on lymphatic endothelial cells. Of these receptors, the roles of RHAMM and CD44 have been most documented for their roles in excisional skin wound repair. Thus, repair of full thickness excisional skin wounds is delayed in RHAMM−/− mice [13] and CD44 is required for efficient response to sterile skin injury [9], keratinocyte proliferation [14] and angiogenesis [29]. Since both of these HA receptors have been reported to bind to HA oligosaccharides, we assessed if they were required for a response to 6mer HA. We therefore compared the effect of 6mer HA applied to wounded RHAMM−/− and CD44−/− mice with wildtype mice as described in Material and Methods. In these experiments, 6mer HA significantly (2 fold) stimulated wound repair in wildtype mice (Fig. 6). In contrast, 6mer HA had no effect on wound repair of RHAMM−/− and CD44−/− mice. These results show that both RHAMM and CD44 are required for a response to 6mer HA.

## Discussion

Collectively, our results show that a 6mer HA oligosaccharide stimulates dermal fibroblast migration in culture, rapid excisional wound closure, increased wound TGFβ1 accumulation and enhanced pro-inflammatory M1 and pro-fibrotic M2 macrophages. However, markers for a subsequent increase in fibrotic markers in wounds were not detected. This apparent inability of the 6mer to stimulate a more robust fibrosis than the PBS control may be due to a loss of 6mer accumulation in the wound over time and a concomitant requirement for its continued presence to provoke later stages of fibrosis. Alternatively the 6mer may be an initiating stimulus that requires the presence of other factors/HA fragments to enhance later stages of fibrotic repair. Nevertheless, our results demonstrate for the first time that the effects of the 6mer are unique and distinct from the other tested HA oligosaccharides and fragments.

Our results also suggest that the 6mer and 8mer are responsible for the stimulating effect that we, and others, have previously reported for mixtures of HA oligosaccharides. Thus, neither the 4mer, 10mer HA oligosaccharides nor HA fragments had an effect on cell migration in scratch wound assays. Native HA inhibited fibroblast migration providing further evidence for the emerging paradigm that native HA has opposing effects to fragmented HA. Although the 6mer and 8mer shared migration promoting properties, the 6mer was unique in its ability to collectively promote wound closure, increase wound M1 and M2 macrophages and increase wound TGFβ1. Furthermore, our results identify the 10mer and 40 KDa fragments as inhibitors of early wound closure. Although it has been shown previously that HA fragments modify wound repair by stimulating angiogenesis, inflammation, cell migration and proliferation until the present study, it was unclear whether a range of HA fragment/oligosaccharide sizes were responsible for stimulating migration, wound closure et cetera or whether these functions were limited to specific sizes of HA polymers. Collectively, our data support a model for unique bio-information residing within specific sizes of HA oligosaccharides and fragments. Intriguingly, different HA polymer sizes appear to share some but not all functions, for example both the 6mer oligosaccharide and the 40 kDa fragment significantly stimulate M1 macrophage accumulation in wounds, and the 6mer and 8mer share an ability to increase fibroblast migration. However, only the 6mer had an effect on TGFβ1 accumulation. If this type of selective sharing of functions is characteristic of other HA sizes, the collective pool of HA fragments within wounds could provide selective signal amplification sufficient for fueling final stages of fibrotic repair.

Adult skin wounds accumulate a wide size range of HA fragments [8]. These fragments are the result of enzymatic and reactive oxygen/nitrogen species driven degradation [30], [31]. For example, platelets are a significant source of Hyal2 during the early stages of wound repair. HA fragments then activate the innate immune response, causing production of cytokines and chemokines such as IL-6 and IL-8, which also provoke further wound infiltration by immune cells and macrophages [5]. HA fragments stimulate migration and differentiation of endothelial cells, thereby contributing to angiogenesis, which is another important aspect of wound repair [19], [32] and also increase migration and proliferation of dermal fibroblasts and keratinocytes. Taken together, HA fragments stimulate many wound repair associated processes that are essential for fast and efficient wound recovery. However, many of these HA fragment-induced effects result in robust fibrosis that can lead to scar formation. Our results suggest that application of specific sizes of HA oligosaccharides/fragments can be utilized to control the balance between wound repair efficiency and quality. Thus, use of 6mer HA to increase wound closure without significantly increasing fibrosis is potentially useful for treatment of delayed or aberrant wound repair.

HA interacts with HA binding proteins, which are located on the cell surface of several cell types and play important roles during wound repair. Dermal fibroblasts, keratinocytes, endothelial cells and macrophages all express HA receptors and can be activated by HA fragments [30], [31]. Although RHAMM/HMMR, CD44 and TLR2/4 all bind HA, the binding affinity for specific HA size ranges differs between receptors. RHAMM/HMMR binds low MW HA with higher affinity compared to CD44, which requires multivalent interactions with HA and therefore has a preference to bind high MW HA [33], [34]. TLR2/4 preferentially binds to small HA fragments such as 6mer HA [35]. The overall effect of HA on cell behavior therefore depends on cell type, HA receptor expression profile as well as the molecular weight distribution of HA. For example, migration and proliferation of keratinocytes is stimulated by medium sized (166 kDa) HA whereas low MW HA (5–20 kDa) and high MW (1090 kDa avg) have no effect [16]. In contrast, a mixture of 2–10mer HA fragments has been shown to increase epidermal thickness by increasing progenitor cell characteristics in the basal cell layer and cell differentiation in a skin equivalent model using human skin derived fibroblasts and keratinocytes [36]. The 6mer HA stimulated wound closure requires the expression of RHAMM−/− and CD44−/− and is consistent with previous reports noting that both receptors are activated by HA oligosaccharides [19], [37]. These results do not exclude the possible involvement of other receptors such as TLR2,4 or LYVE1 but their more restricted expression on cells responding to excisional injury predict they play a more limited role in repair.

6mer HA has previously been implicated in endothelial cell proliferation and tube formation in culture, which are important aspects of angiogenesis *in vivo* [38], [39]. We did not observe an increase of smooth muscle actin positive and therefore mature blood vessels in 6mer HA treated wounds compared to control wounds that have been treated with collagen I matrix only or wounds that have been treated with 40 kDa HA. Activation of TLR4 by small HA fragments also has been implicated in activation of innate immune response, cytokine/chemokine production and wound repair associated inflammation [35]. Infiltration of pro-inflammatory M1 and regulatory M2 macrophages has been increased in 6mer and 40 kDa HA treated wounds compared to wounds that have been treated with collagen I matrix alone. This observed increase in macrophage infiltration in 40 kDa HA treated wounds could be caused by small HA fragments that are present in the 40 kDa HA preparation or that are produced by degradation of 40 kDa HA during wound repair.

The lack of ability of the 6mer or 40 kDa HA fragment to support a robust wound fibrosis could be due to loss of these polymers from the wound site. For example, multiple rather than a single application of HA fragments/oligosaccharides may be required to promote increased neo-angiogenesis, myofibroblast differentiation and collagen production. Alternatively, a 6mer HA oligosaccharide may not stimulate additional processes that are required for these processes. Potentially the presence of other HA oligosaccharides in wounds, such as 10–20mers [18] and larger fragments may be required for progression through the inflammatory to fibrosis to resolution stages of repair. Also, TGFβ induced *in vitro* myofibroblast differentiation is not supported by exogenous HA but requires endogenous HA synthesis [27], [40]. Therefore addition of exogenous HA to wounds may not be sufficient to induce myofibroblast differentiation.
